# Multimodal Imaging Characteristics and Diagnostic Approach to Ancient Schwannoma in a Pediatric Patient

**DOI:** 10.7759/cureus.11003

**Published:** 2020-10-17

**Authors:** Bridget E Ebert, Timothy Singewald, Brianne B Roby, Sivakumar Chinnadurai

**Affiliations:** 1 Otolaryngology, University of Minnesota, Minneapolis, USA; 2 Radiology, Children's Minnesota, Minneapolis, USA; 3 Pediatric Otolaryngology and Facial Plastic Surgery, Children's Minnesota, St. Paul, USA; 4 Pediatric Otolaryngology and Facial Plastic Surgery, Children's Minnesota, Minneapolis, USA

**Keywords:** schwannoma, pediatric radiology, pediatric otolaryngology

## Abstract

Ancient schwannoma is an extremely rare benign, peripheral nerve sheath tumor. Despite its benign nature, its characteristic heterogeneous appearance and degenerative changes commonly lead to misdiagnosis of malignancy. Although schwannomas are extremely uncommon in the pediatric population, these neoplasms have been associated with underlying conditions such as neurofibromatosis type two, and appropriate recognition is important to ensure close monitoring of potential future symptoms secondary to other tumors. We report the imaging and laboratory findings of an ancient schwannoma of the vagus nerve in a 10-year-old female, the first documented case of such a tumor in a pediatric patient, and discuss its characteristic findings and diagnostic considerations. Awareness of this rare tumor can help promote correct diagnosis and avoidance of costly, high-risk diagnostic methods.

## Introduction

Schwannomas are benign, neurogenic tumors originating from Schwann cells surrounding peripheral nerves. These rare lesions can arise from peripheral nerves surrounded by Schwann cells in any area of the body. They are most often found in the head, neck, and extremities [1-3]. The vast majority of schwannomas occur in adults, with fewer than 10% diagnosed in patients younger than 21 years [1-3]. It is relatively common for schwannomas to involve cranial nerves, but schwannomas of the vagus nerve are rare, especially in children, with only 15 cases of pediatric vagal schwannoma reported in literature [1,2].

Schwannomas are divided into different subtypes based on their histologic characteristics. Ancient schwannoma is an uncommon subtype of these rare neoplasms, histologically characterized by degenerative changes and diffuse hypocellular patterning, which often leads to misdiagnosed malignancy despite the tumor’s benign nature [4,5]. There are only a few documented cases of ancient schwannoma in the literature, but the few known cases were most often seen in elderly patients with long-standing tumors [4]. In this report, we describe the first documented case of an ancient vagal schwannoma in a pediatric patient.

## Case presentation

**Figure 1:**
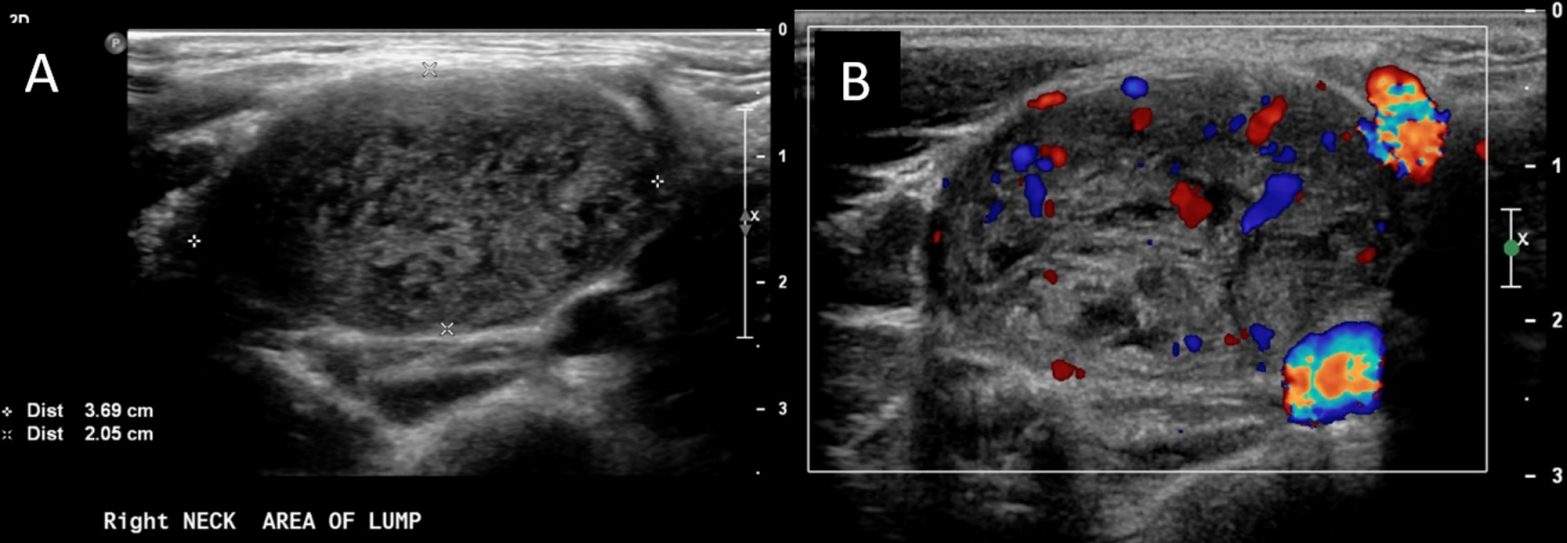
A: Longitudinal ultrasound showing a heterogeneous ovoid mass. B: Internal hypervascularity as demonstrated by Doppler.

**Figure 2:**
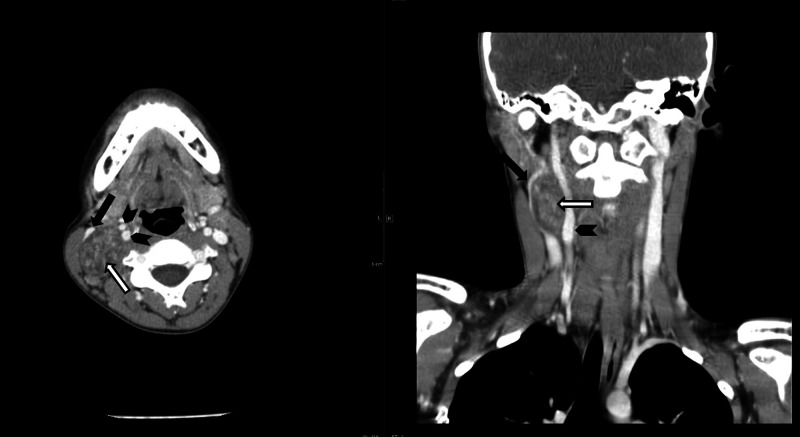
Axial and coronal contrast enhanced CT showing vagal schwannoma (white arrow) next to carotid artery (arrowhead) and compressing internal jugular vein (black arrow). In the axial image, distinction can be seen between the internal and external carotid arteries, supporting the tumor's suspected location within the carotid sheath at the level of the carotid bifurcation.

A 10-year-old female presented to her primary care provider with a three-day history of sore throat and cervical lymphadenopathy. The sore throat improved after four days of antibiotic therapy, but persistent right cervical lymphadenopathy warranted follow-up at her primary care clinic. Aside from periodic low-grade contralateral posterior neck pain and intermittent difficulty in swallowing, the patient was asymptomatic. White blood cell count, mononucleosis spot test, C-reactive protein (CRP), and erythrocyte sedimentation rate (ESR) were unremarkable, and Epstein-Barr virus (EBV) studies were consistent with prior infection. A right neck ultrasound (US) revealed a solid hypervascular mass deep to the right sternocleidomastoid muscle (SCM) and posterior to the carotid artery and internal jugular vein, as seen in Figure 1. A computed tomography (CT) scan was obtained and found a heterogeneously enhancing well-defined mass displaying little surrounding inflammation, reactive prominence of right posterior triangle lymph nodes, and mass effect on right internal jugular vein (Figure 2), suggesting lymph node enlargement but warranting follow-up to rule out a neoplastic process. Based on these results, the patient was directed to present to the Emergency Department at our institution.

**Figure 3:**
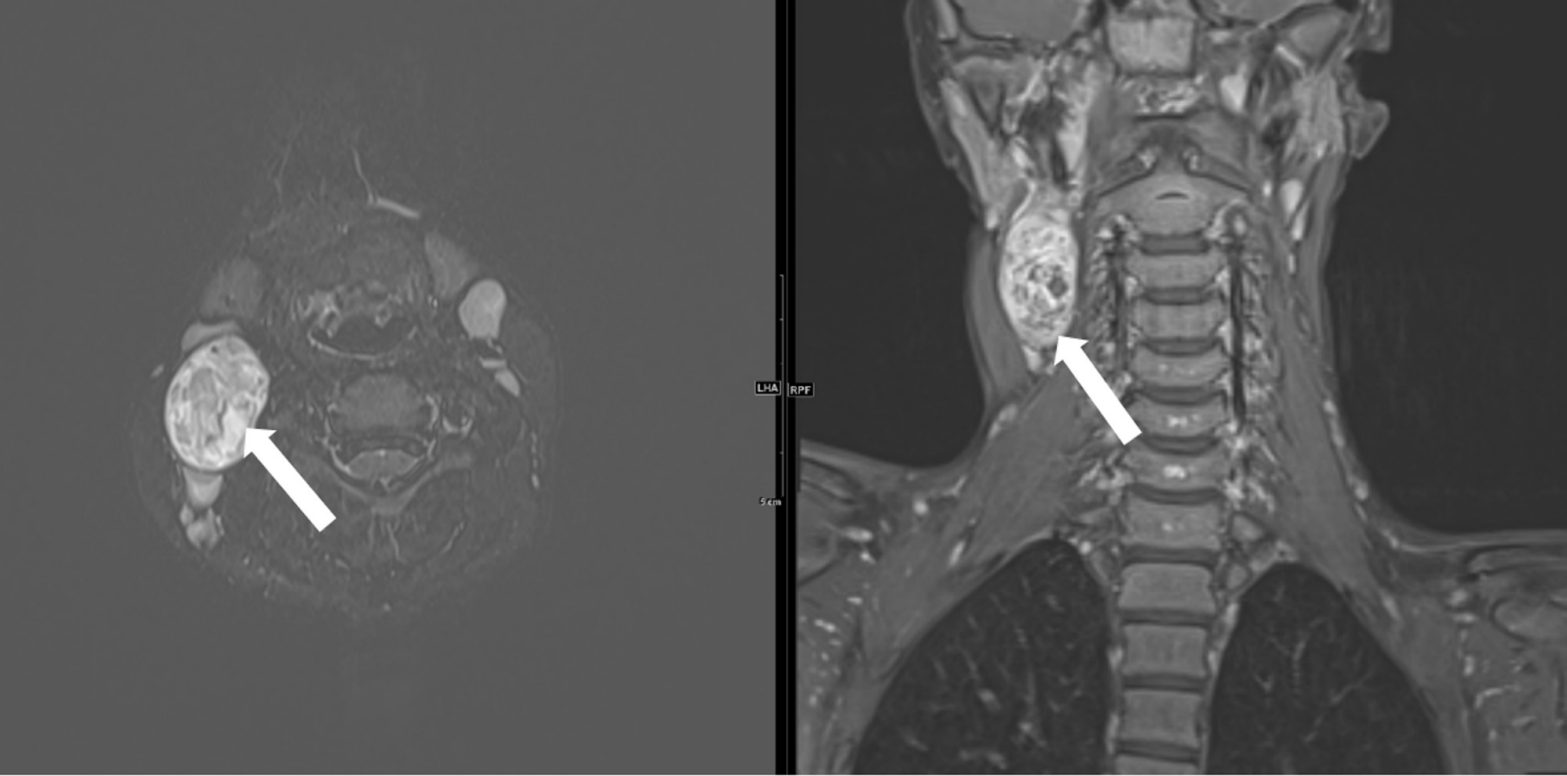
Axial T2-weighted and coronal T1-post contrast MRIs showing vagal schwannoma (arrow) with heterogeneous enhancement and non-enhancing regions suggestive of degeneration.

Outside imaging was reviewed and, after unremarkable CRP, complete blood count, and urine and blood metanephrine studies performed at our institution, magnetic resonance imaging/angiography (MRI/MRA) was obtained. This identified a well-defined 3.0 x 2.3 x 4.1 cm ovoid-appearing, heterogeneous mass seen within the right carotid space. On postcontrast sequences, there was avid enhancement with internal areas demonstrating less prominent enhancement and no restricted diffusion. The mass displaced the right internal jugular vein anterolaterally and the internal carotid artery anteromedially with associated prominent and mildly enlarged cervical lymph nodes felt to be reactive in nature (Figure 3). Given the location and imaging characteristics, a diagnosis of vagal schwannoma was favored. Fine-needle aspiration (FNA) was performed with cytologic and histologic findings consistent with schwannoma, and surgical excision was performed. Anatomic pathology report noted an ancient schwannoma with characteristic atypia and degenerative changes.

## Discussion

Ancient schwannomas are variants of benign peripheral nerve sheath tumors that differ from classic schwannomas based on their histologic degenerative features and nuclear atypia [4,5]. Perivascular hyalinization, calcification, cystic necrosis, and degenerative nuclei are all characteristic features of these neoplasms. These changes are not known to hold prognostic significance; however, they can often lead to a misdiagnosis of malignant soft tissue tumors [5]. Thus, an understanding of the histologic features of this neoplasm as well as their characteristic appearance on imaging is paramount to ensuring a correct diagnosis.

The multimodal imaging (US, CT, MRI/MRA) performed for this patient are illustrative of the imaging characteristics of ancient schwannomas and makes this case unique in comparison to its counterparts which classically include only one or two imaging modalities. US in Figure 1A shows a heterogeneous ovoid mass. CT scan seen in Figure 2 showed a well-defined mass with enhancement in the areas surrounding degeneration, which are findings consistent with ancient schwannoma [5]. In classic schwannoma, these enhancing degenerative areas may not be observed and a target-like appearance made by fibrocollagenous central enhancing areas and peripheral myxomatous regions may be seen instead. MRI, seen in Figure 3, was consistent with the diagnosis of a well-defined, heterogeneous lesion, and heterogeneous enhancement around cystic areas, and supported the diagnosis of ancient schwannoma over the classic subtype. Hypocellular, disorganized, myxomatous areas known as Antoni B areas show high signal intensity on T2-weighted images while their cellular, organized Antoni A counterparts show low-to-intermediate signal intensity [5,6]. The high-intensity patterning in this MRI suggests the presence of increased hypocellular Antoni B areas with smaller Antoni A areas, perhaps a result of their degeneration to cysts or necrosis [5]. These findings are characteristic of ancient schwannomas and are not usually seen in classic schwannomas. Furthermore, this lesion lacks the classic “salt and pepper” MRI appearance classically seen in paragangliomas [7]. CT and MRI both show evidence of anterolateral internal jugular vein displacement and anteromedial carotid displacement consistent with a mass within the carotid sheath. This supports evidence of a vagal over a cervical sympathetic chain lesion, which tends to displace both the internal jugular vein and carotid without separating them.

Although imaging can assist in the diagnosis of ancient schwannoma, histopathology is the only method of confirmation. In this case, diagnosis of schwannoma was made via ultrasound guided FNA and ancient subtype was confirmed post-excision. FNA was chosen to aid in diagnosis, triage urgency of surgical excision, and could be performed with local anesthetic only. The use of FNA in schwannoma diagnosis remains controversial due to challenges in representative specimen acquisition and difficulties in making a confident diagnosis based on a limited sample [2,8]. Despite these potential challenges, FNA was instrumental in the workup for accurate surgical planning and provided the diagnosis in a minimally invasive fashion.

Neurogenic tumors make up only 2% of benign, pediatric neoplasms but they are commonly associated with underlying conditions such as neurofibromatosis types one and two (NF-1, NF-2) [3,6]. Schwannomas in particular are characteristic of NF-2, and while bilateral vestibular schwannomas are the hallmark of the disease, schwannomas of other peripheral nerves may arise and warrant further testing. Cases such as this which involve atypical, faster-growing schwannomas in a pediatric patient support subsequent close monitoring as well as genetic workup.

## Conclusions

Despite the rarity of schwannomas in the pediatric population, clinicians should be aware of their presence due to the association with underlying genetic conditions and symptoms related to those conditions. FNA should be strongly considered to aid in diagnosis and accurate surgical planning and can be performed safely with minimal to no sedation. Understanding the histologic and radiologic characteristics of the ancient schwannoma subtype can help avoid misdiagnosis of malignancy and improve efficiency and accuracy of care.
